# Anomalous self-experiences in biotypes of psychosis

**DOI:** 10.1007/s00406-026-02257-3

**Published:** 2026-06-05

**Authors:** Luis Sobrino-Conde, Antonio Arjona-Valladares, Emma Osorio-Iriarte, Rosa Beño-Ruiz de la Sierra, Claudia Rodríguez-Valbuena, Juan Carlos Fiorini-Talavera, Marta Hernández-García, Vicente Molina

**Affiliations:** 1 Psychiatry Service, University Clinical Hospital of Valladolid, Valladolid, Spain; 2 Psychiatry Department, School of Medicine, University of Valladolid, Valladolid, Spain; 3 Biomedical Research Institute of Valladolid (IBioVall), Valladolid, Spain

**Keywords:** Anomalous self-experiences, Biotypes, Psychosis, Schizophrenia

## Abstract

Biotypes of psychoses represent transdiagnostic clusters based on pathological characteristics relevant to this syndrome. This approach seeks to help with the delimitation of groups with external validity within the psychotic syndrome beyond current categories. Our group has previously proposed two biotypes characterized by different cognitive performance and anatomical and functional alterations. Here, we propose that these groups may also differ in the presence and/or severity of anomalous self-experiences. We included 113 patients with psychotic disorders, classified as biotypes into clusters 1 (*n* = 30, with severe cognitive deficit) and 2 (*n* = 83, with moderate cognitive deficit) according to criteria established in previous studies (Comparelli et al in Compr Psychiatry 65:44-49, 2016) and (Nelson et al in Schizophr Res 152:20-27) healthy controls. The Inventory of Psychotic-Like Anomalous Self-Experiences (IPASE) was used to score anomalous self-experiences. Cluster 1 patients (severe cognitive impairment) showed significantly higher IPASE scores than healthy controls and Cluster 2 patients, whereas differences between Cluster 2 patients and healthy controls were less pronounced. We conclude that severe anomalous self-experiences characterize the biotype 1 of psychoses, alongside marked cognitive deficits, anatomical alterations, functional basal hyperactivity and higher negative symptoms scores.

## Introduction

Anomalous Self-Experiences (ASEs) involve a wide range of experiential deviations that affect the fundamental sense of being an embodied, self-present subject engaged with the surrounding world [26, 30]. These disturbances include both cognitive and somatic aspects, such as alterations in the stream of consciousness and the implicit sense of the naturalness of mental processes, the first-person sense of presence, the sense of corporeality, the self-demarcation at the self-world boundary, and existential reorientation [26]. Thus, ASEs can compromise ipseity, that is, the automatic and pre-reflective experience of individual identity [28].

Previous research clearly supports the increased frequency and severity of ASEs in patients with psychosis [27], even in its early stages [13] and in at-risk states [16], which remain stable over time in this syndrome [24]. Although ASEs have been mostly reported for schizophrenia patients, they can also be observed in cases of bipolar psychosis [13]. Concerning their neurobiological substrates, a significant relation has been reported between the presence and severity of ASEs in schizophrenia and impairment in corollary discharge (CD) [1], a mechanism known to underlie the preconscious identification of self or alien sources of sensory stimulation. The alteration of CD in bipolar patients was intermediate between that of schizophrenia and healthy control subjects [2].

To ascertain the underpinnings of ASEs, it is also relevant to examine the described significant relation between ASEs and general cognition. Anomalies in the experience of self have been linked to cognitive deficits in schizophrenia, showing negative associations with domains such as attention, working memory, visual learning, and reasoning [33]. Furthermore, certain domains of ASEs (e.g., Self-awareness and Presence, and Consciousness) have been inversely linked to specific cognitive abilities such as motor speed and problem-solving [15]. However, this association has not been observed in other studies [7]. Since cognitive alterations are not found in all schizophrenia patients [3], such discrepancies suggest that ASEs may be more severe in or even restricted to a subset of cases with psychoses. In this context, the recent description of biotypes across the psychotic spectrum (including affective and non-affective psychoses) becomes particularly relevant. At least two independent groups have converged on the finding of a transdiagnostic biotype with greater cognitive impairment [6, 8]. Specifically, our group has identified two cognitive subtypes of psychosis characterized by distinct neuropsychological profiles and associated anatomical and functional abnormalities, including structural brain abnormalities and differences in baseline functional activity. The subgroup with more severe cognitive impairment has been associated with greater clinical severity, reduced subcortical volumes -particularly in the thalamus and hippocampus- reduced frontal structural connectivity, and altered patterns of functional brain activity, including baseline hypersynchrony and reduced modulation during cognitive processing, supporting the idea that distinct neurobiological substrates exist in this subgroup [8].

Taken together, these findings suggest that cognitive subtypes within the psychosis spectrum may be associated with differences in the expression and severity of ASEs. Therefore, we hypothesize that ASEs are more severe or even characteristic of this biotype. Within this framework, the present study aimed to investigate differences in anomalous self-experiences between previously defined cognitive biotypes of psychosis and healthy control subjects, to determine whether the severity of ASEs constitutes a relevant feature of the biotype characterized by larger cognitive deficits.

## Subjects and methods

### Sample

This study included 83 individuals diagnosed with schizophrenia (22 experiencing a first-episode (FE) and 61 with a chronic, stable condition), 30 patients with bipolar disorder and 22 healthy controls. Diagnoses were made by two experienced psychiatrists involved in their treatment and following the criteria outlined in the Diagnostic and Statistical Manual of Mental Disorders, Fifth Edition (DSM-5).

Exclusion criteria comprised: (i) presence of neurological disorders, (ii) history of head trauma involving loss of consciousness, (iii) current substance abuse (excluding nicotine and caffeine), (iv) Intelligence Quotient (IQ) below 70, (v) any psychiatric treatment for control participants, and (vi) any current diagnosis other than schizophrenia or bipolar disorder for patient participants.

Sociodemographic, behavioral, cognitive, and clinical characteristics are detailed in Table 1. Current antipsychotic doses were converted to chlorpromazine equivalents in mg/d (Table 1).

**Table 1:** Sociodemographic, clinical and cognitive values in the sample

	HC (*n* = 22)	Cluster 1 (*n* = 30)	Cluster 2 (*n* = 83)
Age	40.7(9.3)	43.6(10.3)	40.1(10.7)
Sex (M/F)	13/9	19/11	63/20
Education (years)	15.1(2.4)	11.1(3.6)## , *	13.7(3.5)
CH SZ/FE/BD		21/4/5	40/18/25
Illness duration (Months)		169.0(154.5)	138.7(135.6)
CPZ equivalents (m/d)		353.7(215.7)	334.5(212.5)
PANSS positive		12.0(4.1)	10.6(4.2)
BNSS total		25.0(15.0)*	17.7(16.6)
PANSS total		59.0(21.8)*	47.5(16.5)
WAIS-Total IQ	108.4(11.3)	84.2(10.4)	99.8(12.4)
BACS-Verbal memory	*47.0(5.1)47.0(5.1)*	*30.3(8.6)###*, ***	*43.3(9.2)*
BACS-Working memory	*21.9(4.5)*	*13.0(3.5)###*, ***	*19.2(3.6)#*
BACS-Motor speed	*75.0(11.6)*	*51.1(12.3)###*, ***	*66.4(14.9)*
BACS-Verbal fluency	*24.4(4.0)*	*16.0(4.3)###*, ***	*23.9(5.2)*
BACS-Processing speed	*62.0(6.2)*	*31.0(5.9)###*, ***	*48.4(11.8)###*
BACS-Problem solving	*18.0(2.9)*	*12.5(4.3)###*, ***	*17.9(2.3)*
WCST-Perseverative errors (%)	*9.8(3.8)*	*23.1(12.0)###*, ***	*11.4(6.5)*
IPASE-Cognition	9.3(2.9)	16.9(8.0)###, *	12.9(5.6)##
IPASE- Self-awareness and Presence	32.6(9.4)	56.2(20.6)###, *	45.0(17.7)
IPASE-Consciousness	12.2(4.6)	17.1(6.5)##, *	13.8(5.2)
IPASE-Somatization	27.9(11.5)	40.1(14.5)###	35.2(11.9)#
IPASE- Demarcation/Transitivism	7.1(2.3)	10.8(5.0)##	9.6(4.1)#
IPASE-Total ASEs	89.1(25.0)	140.6(50.0)###, *	116.4(39.4)#

Data are given as mean (standard deviation)

Statistical comparisons were performed using Kruskal–Wallis tests for continuous variables and χ² tests for categorical variables. When significant group effects were detected, pairwise comparisons were performed using Mann–Whitney U tests with Bonferroni correction

*M/F* Masculine/Feminine, *CH SZ* Chronic schizophrenia, *FE* First psychotic episode, *BD* Bipolar disorder, *CPZ* Chlorpromazine, *PANSS* Positive and negative syndrome Scale, *BNSS* Brief negative symptom scale, *WAIS* Wechsler adult intelligence scale, *IQ *Intelligence quotient, *BACS* Brief assessment of cognition in schizophrenia, *WCST* Wisconsin card sorting test, *IPASE* Inventory of psychotic-like anomalous self-experiences, *ASEs* Anomalous self-experiences

Significance levels: # p < 0.05; ## p < 0.005; ### p < 0.0001 (Patients vs Controls); *p < 0.05,***p<0.001 (Cluster 1 vs. Cluster 2)

All participants provided written informed consent after receiving comprehensive printed information. The study was approved by the ethics committees of the Clinical Hospital of Valladolid (protocol PI-21–2623).

#### Cognitive assessment

 Cognition was assessed with the Spanish version of the Brief Assessment of Cognition in Schizophrenia (BACS) [32], which measures verbal memory, working memory, motor speed, verbal fluency, attention, processing speed, executive function, and problem-solving. In addition, the Wisconsin Card Sorting Test [4](WCST; percentage of perseverative errors) was used, which measures performance in cognitive flexibility, abstract reasoning, and response inhibition. Global Intelligence Quotient (IQ) was evaluated with the Wechsler Adult Intelligence Scale III [9].

#### Anomalous Self-Experiences (ASEs) assessment

ASEs were assessed using the Inventory of Psychotic-Like Anomalous Self-Experiences (IPASE) [5], a 57-item self-report questionnaire structured around five factors. Participants completed the scale in the presence of a researcher, indicating their level of agreement with each statement on a scale from 1 (Strongly Disagree) to 5 (Strongly Agree). Thus, the minimum score is 57 (indicating absence of ASEs).

- The Cognition factor includes items addressing disruptions in thought processes, such as thought interference.
- The Self-awareness and Presence factor captures experiences of diminished self-identity and a weakened sense of connection with the external world.
- The Consciousness factor reflects changes in the perception of time, intentionality, and difficulty distinguishing imagination from reality.
- The Somatization factor pertains to altered bodily experiences, such as feeling the body has changed shape, is difficult to control, or a sense of physical or psychological absence from one’s own body.
- The Demarcation/Transitivism factor involves disturbances in the boundaries between self and environment, or a sense of nonexistence.

#### Cluster definition

Classification of patients into clusters was based on criteria reported in our previous definition of transdiagnostic biotypes [8]. From the sample included in that study, only cases with IPASE scores were used for the present analysis.

In summary, their classification utilized Brief Assessment of Cognition in Schizophrenia (BACS) subscales and the percentage of perseverative errors in the Wisconsin Card Sorting Test (WCST). *All test scores were standardized to ensure equal weighting across cognitive measures prior to factor analysis to derive individual cognitive profiles*. Using K-means clustering, silhouette analysis, and hierarchical methods, two distinct clusters were identified, where each case was assigned to the corresponding cluster depending on its distance to the cluster centroid [8]:

- Cluster 1: characterized by severe cognitive impairment, along with hyperactive baseline neural activity and anatomical abnormalities in the thalamus, hippocampus, cortical thickness, mean fractional anisotropy and higher negative symptoms scores.
- Cluster 2: characterized by milder cognitive deficits and less pronounced functional and anatomical alterations.

According to this method, in the present sample 30 patients belonged to Cluster 1 (including 21 with chronic schizophrenia, 4 with FE and 5 with bipolar disorder; 19 males, 11 females) and 83 patients to Cluster 2 (40 with chronic schizophrenia, 18 with FE and 25 with bipolar disorder; 63 males and 20 females). Scores from the 22 HC were used as a reference.

### Statistical analysis

In a first step, we assessed the significance of the differences in age and sex distribution between groups (Clusters 1 and 2, and HC) using Kruskal-Wallis and Chi squared tests, respectively. Similarly, illness duration and treatment doses were compared between clusters using Mann-Whitney U tests.

The main hypothesis of the study was tested by comparing total IPASE scores between HC, and patients in Clusters 1 and 2. The statistical significance of these differences was assessed with the Kruskal-Wallis test since homoscedasticity of the distribution of those scores was not met, according to the Levene test. If a significant group effect was observed in that test, a second Kruskal-Wallis test was planned to examine the differences in each of the IPASE subscales. Significance levels were adjusted using the Bonferroni correction (6 scores, *p* = 0.0083).

Then, follow-up analyses with Mann-Whitney U tests were used to assess the significance of the differences between each pair of groups in total and subscales scores of IPASE. Effect sizes of the differences were calculated as $\:r=Z/\sqrt{N}$, where 0.1 corresponds to a small effect size, 0.3 to a middle effect and 0.50 to a large effect.

Finally, to rule out potential confounders, we examined the significance of the relations between IPASE scores and illness duration and, on the other hand, with antipsychotic doses (in chlorpromazine equivalents (CPZ)/day) and age, using Spearman’s correlation coefficients.

## Results

Age (Χ^2^ = 2.19, df = 2, *p* = 0.299) and sex (Χ^2^ = 1.86, df = 2, *p* = 0.394) did not differ significantly between groups. Illness duration and treatment doses were not significantly different between clusters. Illness duration (U = 871, z = 1.01, *p* = 0.29) and antipsychotic dose (U = 1061, z = 0.57, *p* = 0.57) did not differ between clusters.

**Fig. 1:**
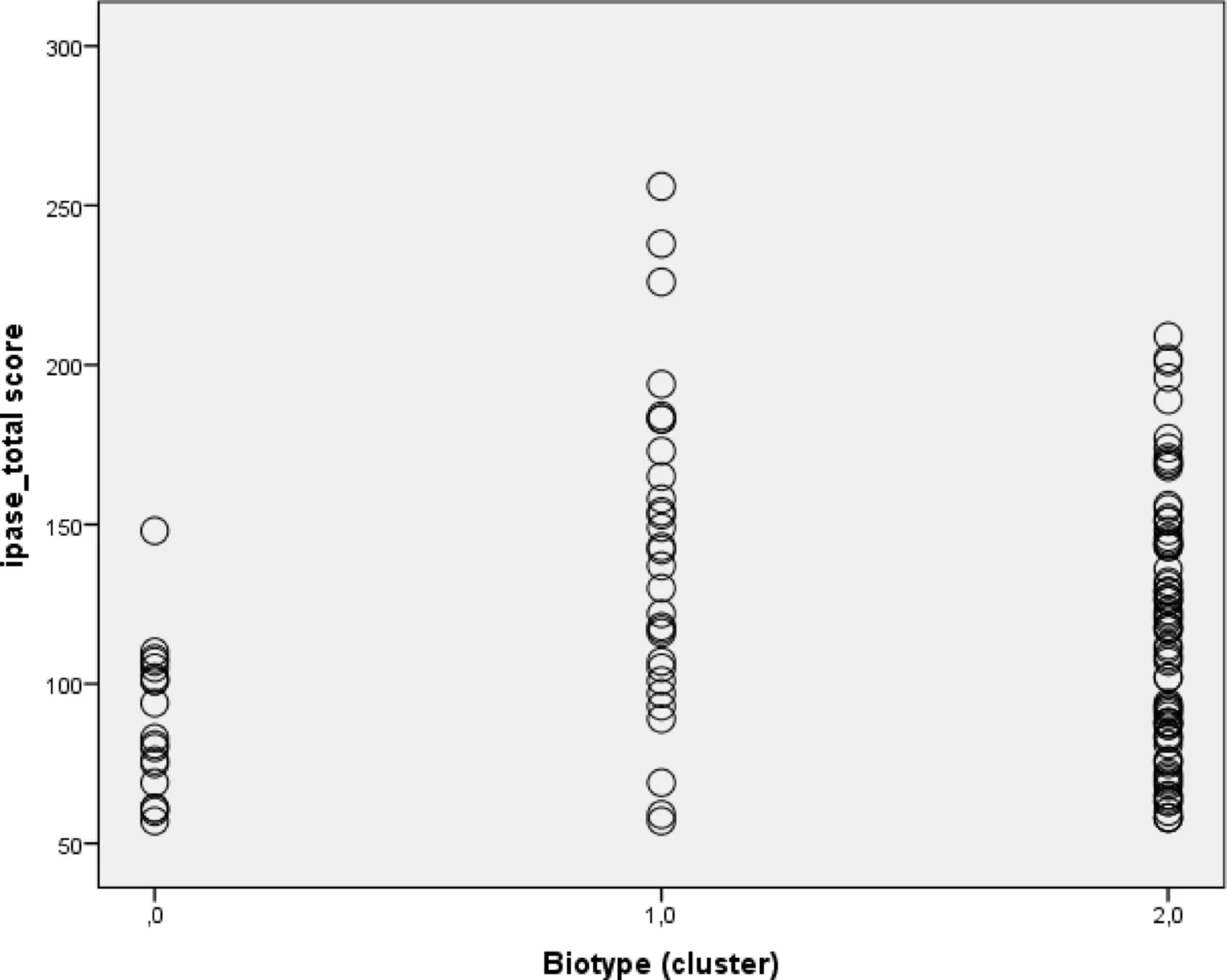
Distribution of individual total scores between clusters (0 = healthy controls; 1 = severe cognitive deficit; 2 = moderate cognitive deficit)

Total IPASE scores were significantly different among groups (Χ^2^ = 17.17; df = 2; *p* < 0.001; Fig. 1). Similarly, scores in Cognition (Χ^2^ = 13.08; df = 2; *p* < 0.001), Self-awareness and Presence (Χ^2^ = 20.73; df = 2; *p* < 0.001), Consciousness (Χ^2^ = 8.34; df = 2; *p* = 0.015), Somatization (Χ^2^ = 11.15; df = 2; *p* = 0.004) and Demarcation/Transitivism (Χ^2^ = 9.72; df = 2; *p* = 0.008) showed a significant effect of group.

In follow-up comparisons, Cluster 1 patients showed significantly higher scores than HC in total IPASE (U = 120; z = 3.88; *p* < 0.001; *r* = 0.54), as well as in Cognition (U = 148.5; z = 3.41; *p* < 0.001, *r* = 0.47), Self-awareness and Presence (U = 105.5; z = 4.16; *p* = 0.001; *r* = 0.57), Consciousness (U = 171; z = 2.97; *p* = 0.003; *r* = 0.41), Somatization (U = 151; z = 3.32; *p* = 0.001; *r* = 0.46) and Demarcation/Transitivism (U = 186.5; z = 2.66; *p* = 0.008; *r* = 0.37) subscales.

Similarly, Cluster 1 patients showed higher scores than Cluster 2 patients in total IPASE scores (U = 896.5; z = 2.25; *p* = 0.02; *r* = 0.21) and in Cognition (U = 936; z = 2.02; *p* = 0.04; *r* = 0.19), Self-awareness and Presence (U = 830.5; z = 2.69; *p* = 0.007; *r* = 0.25) and Consciousness (U = 896.5; z = 2.27; *p* = 0.02; *r* = 0.21) subscales. However, scores in Somatization (U = 1031; z = 1.38; *p* = 0.16) and Demarcation/Transitivism (U = 1071; z = 1.13; *p* = 0.26) subscales of the IPASE were not significantly different between patient clusters.

Cluster 2 showed significantly higher scores than HC in total IPASE (U = 590; z = 2.57; *p* = 0.01; *r* = 0.25) and in Cognition (U = 516.5; z = 3.12; *p* = 0.002; *r* = 0.34), Self-awareness and Presence (U = 752; z = 3.12; *p* = 0.001; *r* = 0.31), Somatization (U = 583; z = 2.60; *p* = 0.009; *r* = 0.25) and Demarcation/Transitivism (U = 587; z = 2.59; *p* = 0.01; *r* = 0.25) subscales. Scores in the Consciousness subscale were not significantly different between these groups (U = 752; z = 1.27; *p* = 0.204).

The correlations between ASEs scores (total and subscales) and medication doses and between ASEs and illness duration were not significant (rho < 0.15 in all cases, p = ns).

## Discussion

According to our results, severe ASEs could characterize a biotype within the psychotic syndrome with greater cognitive impairment, which would include a large proportion of schizophrenia and a minority of bipolar patients. This biotype corresponds to Cluster 1 in the present study. To our knowledge, this is a novel result that may contribute to assessing the substrates of those experiences and their relation to psychotic syndromes.

The HC in our study showed mild IPASE scores, supporting the presence with mild severity of ASEs in subjects of the general population. Similar scores have been reported in HC by other groups using the same instrument [22, 31]. Healthy samples in other studies were also found to score mildly in ASEs [17], in this case using the Examination of Anomalous Self-Experience (EASE) [25]. Mild ASEs in the general population can be expected since they include non-necessarily pathological experiences, such as depersonalization feelings or existential preoccupation, among others. Moreover, some ASEs are similar to subclinical psychotic experiences described in this population in dimensional continuum with clinical groups [34].

Therefore, it is the severity of these experiences, and not their mere presence, which discriminates between biotypes. The greater severity of ASEs in Cluster 1 is coherent with the higher self-disturbance scores in FE of schizophrenia as compared to FE of other psychoses [20], and with the milder severity of ASEs in bipolar disorder [11], considering the higher proportion of schizophrenia in cluster 1 and bipolar cases in Cluster 2.

IPASE scores in psychotic patients are related to cognitive performance [15], consistent with the larger cognitive deficit in the biotype Cluster 1 [8]. In the same line, verbal memory deficits have been associated with high levels of ASEs [12, 29]. However, other studies failed to find such a relationship between ASEs and cognition [7, 23]. A possible explanation for this discrepancy would be that samples with higher numbers of cases with smaller cognitive deficit could not show a significant relation between general cognition and ASEs. Thus, depending on the proportion of patients with severe cognitive deficit in the samples, the relationship may be weaker.

The self-feeling or self-experience likely requires an integrated widespread network, as proposed, among others, in the theory of neuro-matrix [18]. Cluster 1 is characterized, in addition to severe ASEs, by basal hyperactivation, volume deficits in thalamus and hippocampus, thinner cortex and lower fractional anisotropy value [8]. These alterations may contribute to a decreased capacity for global integration likely necessary for a preserved self-experience and thus may predispose Cluster 1 patients to more severe ASEs than Cluster 2. A previous report not overlapping with the present sample showed a significant relation between ASEs and basal overconnectivity [14], and it has been also shown that such hyperactivity hampers adequate modulation of the global network [10]. Therefore, hyperconnectivity in Cluster 1 patients could hamper their adequate modulation of cortical networks underlying ipseity and render them more prone to severe ASEs.

Furthermore, we have reported a relationship between ASEs and dysfunction in the corollary discharge mechanism [1], which is however not necessarily restricted to Cluster 1 patients. This neural mechanism is crucial for recognizing the source of actions as self-generated [21], thus playing a vital role in self-identification. Consequently, its dysfunction is likely to represent a neural signature of altered ipseity. In line with this, previous work has described accentuated sensorimotor reactivity, as reflected in neural responses to stimulus salience, in a psychosis cluster characterized by marked, although less severe, cognitive impairment than the cluster showing the most profound and generalized cognitive deficits [4]. The presence of ASEs in Cluster 2 patients suggests the possibility of different biological mechanisms contributing to it, given the significant differences in brain structure and function between these clusters [8]. Together, these findings raise the possibility that similar experiential disturbances may arise from different underlying pathophysiological pathways across biotypes.

Concerning possible sources of error, ASEs may in this context not be a simple effect of chronicity, since some FE patients fell in Cluster 1, consistent with reports showing ASEs in FE samples [13, 35]. In the same line, illness duration did not differ between groups, and no significant relationship was found between ASEs and that duration. Cluster 1 patients were significantly less educated, but this is to be expected given the poorer real-life adjustment in several domains of Cluster 1 patients after 3 years of follow-up [19].

Among limitations, our sample is relatively small. It is possible that a larger sample size could allow identifying a third biotype without cognitive impairment, as in [6], where ASEs could be hypothesized not to be significantly different as compared to HC. Although we have not included patients without treatment, our correlation analyses support that differences in ASEs between biotypes are not an effect of chronicity or antipsychotic treatment. We used the IPASE, but the gold-standard instrument for the evaluation of self-experiences is the Examination of Anomalous Self-Experience (EASE) [25]. Nevertheless, scores using both instruments show a high correlation [22] and a researcher was present to assist the subject in case of poor understanding of item phrasing. It is possible that a similar exam using the EASE could identify ASEs with greater discriminatory capacity between biotypes.

## Data Availability

Data area available on request to the corresponding author.
